# Role of Sirtuins in Tumor Angiogenesis

**DOI:** 10.3389/fonc.2019.01516

**Published:** 2020-01-17

**Authors:** Lincy Edatt, Aswini Poyyakkara, Grace R. Raji, Vishnu Ramachandran, S. Sharath Shankar, V. B. Sameer Kumar

**Affiliations:** Department of Biochemistry and Molecular Biology, Central University of Kerala, Kasaragod, India

**Keywords:** sirtuins, tumor angiogenesis, metabolism, histone deacetylases, endothelial cells, signaling pathways

## Abstract

Generally, changes in the metabolic status of cells under conditions like hypoxia and accumulation of lactate can be sensed by various sensing mechanisms, leading to modulation of a number of signal transduction pathways and transcription factors. Several of the proangiogenic cytokines like VEGF, FGF, PDGF, TGF-β, Ang-2, ILs, etc. are secreted by cancer cells, under hypoxic microenvironment. These cytokines bind to their receptors on the endothelial cells and activates a number of signaling pathways including Akt/PIP3, Src, p38/MAPK, Smad2/3, etc., which ultimately results in the proliferation and migration of endothelial cells. Transcription factors that are activated in response to the metabolic status of tumors include HIFs, NF-κb, p53, El-2, and FOXO. Many of these transcription factors has been reported to be regulated by a class of histone deacetylase called sirtuins. Sirtuins are NAD^+^ dependent histone deacetylases that play pivotal role in the regulation of tumor cell metabolism, proliferation, migration and angiogenesis. The major function of sirtuins include, deacetylation of histones as well as some non-histone proteins like NF-κB, FOXOs, PPAR⋎, PGC1-α, enzymes like acetyl coenzymeA and structural proteins like α tubulin. In the cell, sirtuins are generally considered as the redox sensors and their activities are dependent on the metabolic status of the cell. Understanding the intricate regulatory mechanisms adopted by sirtuins, is crucial in devising effective therapeutic strategies against angiogenesis, metastasis and tumor progression. Keeping this in mind, the present review focuses on the role of sirtuins in the process of tumor angiogenesis and the regulatory mechanisms employed by them.

## Introduction

Angiogenesis, the process of formation of new blood vessels from pre-existing ones, is essential for the normal growth, development and wound healing. Apart from this, angiogenesis is also inevitable for tumor growth and metastasis (1–5). The expression and secretion of various modulators of angiogenesis is regulated by microenvironmental factors like hypoxia and accumulation of different metabolites (6–8). Under conditions like hypoxia, a number of signal transduction pathways and transcription factors like PPARα, PGC-1α, AMPK, FOXOs, etc. gets activated (9, 10). The expression and activation of these transcription factors has been reported to be regulated by a class of histone deacetylase called sirtuins or SIRT (11, 12). Sirtuins are NAD^+^ dependent histone deacetylases that play a vital role in the regulation of metabolism, aging, oncogenesis, angiogenesis and cancer progression (13, 14). It has been reported that SIRT1 can function as a redox sensor, and its activity might be dependent on the overall metabolic status of the cell (15), since it has been shown to regulate the stabilization of transcription factors such as HIF1α under hypoxic conditions (16). Therefore, understanding the regulatory mechanisms employed by sirtuins to modulate tumor angiogenesis is essential for developing effective anti-cancer and anti-angiogenic therapeutic strategies.

## Sirtuins: Mechanism of Action and Classification

In mammals, seven homologs of sirtuins, i.e., SIRT1–SIRT7 (17, 18) which were initially described as class III HDACs (Histone deacetylases), are now known as class III KDACS (Lysine deacetylases) (19). The proposed mechanism of Sirtuin deacetylation is reported to be ADPR-peptidyl-imidate (20) where, Sirtuins catalyze NAD^+^-dependent deacetylation of acetyl lysine, producing nicotinamide, deacetylated lysine, and 2′-O-acetyl-ADP-ribose (21). The major function of sirtuins involve, removal of acetyl groups from the acetyl lysine-modified proteins (22, 23). The reaction gets initiated when, NAD^+^ binds to the catalytic site of sirtuin, with the C1 of NAD^+^ getting placed at the channel junction that, houses the acetyl lysine (24). To understand how increasing levels of NAD^+^ affects sirtuin activity, NAD^+^ synthesis was enhanced by supplementing different precursors for NAD^+^ like nicotinic acid to Preiss-Handler pathway and the result showed increased activation of sirtuins and other enzymes which are NAD^+^ dependent (25). Cellular [NAD^+^]/[NADH] ratio is reported to control deacetylase activity of the sirtuins where, NAD^+^ works as activator, and both nicotinamide and reduced nicotinamide adenine dinucleotide (NADH) acts as inhibitors (26, 27). Sirtuins (SIRT1-3, 5, and 7) catalyze deacetylation reaction on lysine residues of target proteins (28) whereas, SIRT4 and SIRT6 catalyze ADP-ribosylation reaction, by transferring ribosyl moiety to the substrates (29). Sirtuins carry out transcriptional repression where acetylated histones H1, H3, and H4 act as substrates (30). In addition, a number of non-histone proteins like nuclear factor-κB (NFκB), forkhead box type O transcription factors (FOXO), peroxisome proliferator-activated receptor ⋎ (PPAR⋎), coactivator 1α (PGC-1α), enzymes like acetyl coenzyme A (CoA) synthetase 2 (AceCS2), and structural proteins, such as α-tubulin are also deacetylated by sirtuins (29). In contrast to other KDACs, whose only function include deacetylation (31), sirtuins can also remove other groups like glutaryl (32), crotonyl (33), succinyl (34), palmitoyl (35), and myristoyl (36) groups. SIRT1-3 is reported to deacylate hydrophobic (butyryl group) and SIRT5, acidic acyl group (Malonyl group) in histones (37, 38). Also, some non-histone proteins like IDH2, MnSOD and TNFα have been reported to be deacylated by sirtuins (39). It is observed that SIRT4 has both, deacylase activity in leucine metabolism and lipoamidase activity in decarboxylation of pyruvate, to generate acetyl CoA (40, 41). The intracellular distribution of sirtuins differs. While SIRT1, 6 and 7 are located within the nucleus, SIRT2 is located in the cytoplasm and SIRT 3, 4, and 5 are located within mitochondria (42). In addition, SIRT1 and SIRT3 are known to shuttle to cytoplasm and nucleus, respectively (43, 44). These findings establish sirtuins as important players in epigenetic gene regulations.

## Sirtuins in Endothelial Cell Functions

Different classes of sirtuins have been widely studied in endothelial cell growth and maintenance (45–47). While, blocking the function of SIRT1 reduced endothelial sprout formation, migration and the assembly of primitive vascular network (14) it was observed that, knockdown of SIRT1 altered the levels of sprouting angiogenesis due to reduction of MMP14 expression (48, 49). Potente et al. reported that SIRT1 deacetylates FOXO1, a negative regulator of angiogenesis, as SIRT1- deficient ECs showed abnormal angiogenic behavior due to FOXO1 activity (14). Nutrient deprivation and cellular energy shortage increase the levels of NAD+ and thus the expression and activation of sirtuins (50). It was observed that, endothelial tip cells employ anaerobic glycolysis for generating ATP (Warburg effect) rather than oxidative phosphorylation (51). In addition to promoting endothelial cell proliferation and angiogenesis, this makes ECs more resistant to hypoxia too (52, 53). Recent studies have established that SIRT1 modulates tip and stalk behavior through deacetylation of intracellular domain (NICD) of NOTCH1 in tumor associated endothelial cells (54). Interestingly, sirtuins also regulate, endothelial homeostasis by modulating the endothelial nitric oxide synthase (eNOS) (55). Recent studies reveal that endothelial SIRT1 deficiency, causes fibrosis due to aberrant secretion of ligands of Wnt and Notch pathways, as well as proteolytic fragments of glycocalyx core protein (56). Some studies also reported that SIRT1 can mediate transcriptional repression in association with Hey2 and Hes1 during vascular development (57, 58). In ECs, over expression of SIRT1 prevents cellular senescence, enhances vasodilatory responses, and alleviates aging-induced vascular impairment (59, 60) whereas, SIRT1 deficiency results in reduced migration in response to chemoattractant (14). In addition to SIRT1, SIRT2 is reported to regulate the survival and energy metabolism of ECs. Studies by Zhang et al. demonstrated that SIRT2 inhibition reduce the survival rate of PIEC cells as it causes mitochondrial depolarization (61). SIRT2 is also reported to promote Ang II-induced cytoskeletal remodeling in ECs (62). In addition, SIRT2 knock down studies revealed altered expression of migration associated genes like CALD1 (caldesmon) and CNN2 (calponin) (63, 64). SIRT3 was observed to increase survival of ECs especially during hypoxia through elevating the levels of deacetylation of FOXO3 (65). Recent studies also revealed that in SIRT3 deficient endothelial cells the expression of PFKFB3 was downregulated causing attenuation of glycolysis and angiogenesis (66). SIRT4 however appears to inhibit mononuclear cell adhesion to pulmonary microvascular endothelial cells through repression of E-selectin and VCAM-1 (67). While, angiogenic capacity of endothelial progenitor cells were significantly reduced due to down regulation of CXCR4/JAK2/SIRT5 signaling (68), it was observed that, SIRT6 protects endothelial cells from DNA damage and telomere dysfunction (62, 69). Though various members of the SIRT family has been implicated in the regulation of EC biology, the role of SIRT-1 is the most widely studied ones.

## Sirtuins in Tumor Angiogenesis and their Regulatory Strategies

Expression pattern of sirtuins varies in different types of cancers. While, SIRT1, 4, 5, and 7 have been reported to be upregulated in certain cancers (70–72), the same SIRT1as well as SIRT2 and SIRT6 is shown to be downregulated in breast cancer, hepatic cell carcinoma (73), gliomas, gastric carcinomas (74, 75) and colon adenocarcinoma (76). SIRT1 mainly, mediates heterochromatin formation by deacetylation of histone H1 K26, histone H3 K9 and histone H4 K16, thereby causing deacetylation of non-histone proteins, like transcription factors (E2F1, p53, FOXO, BCL6, p53, Rb), DNA repair proteins and signaling factors (77). SIRT1 mediates regulation of gene expression in response to metabolic status by modulating FOXOs (78). Such a deacetylation of FOXOs by SIRT1 alters various signaling pathways, inhibit apoptosis and regulates mechanisms involved in oxidative stress (79, 80). In general, p53 negatively regulates angiogenesis either, by increasing the production of anti-angiogenic factors or inhibiting pro-angiogenic factors (81). SIRT1 has been reported to regulate neovascularization, through reducing the transcriptional activity of p53 by deacetylation of lysine (45, 46, 82). Apart from SIRT1, SIRT3 and SIRT7 has also been reported to deacetylate p53 thus, negating p53 activity (83, 84). SIRT1 also deacetylates other transcription factors like p73, E2F1, SMAD 7, NFKB and modulate apoptosis and inflammatory responses (45, 46, 85). It was observed that resveratrol, a SIRT1 activator reduced total VEGFR2 expression and inhibited phosphorylation of VEGFR2 by VEGF (86). Also, SIRT1 negatively modulates Delta-like ligand 4 (DLL4)/Notch pathway, inactivates elongation factor2 through activation of ELF2kinase and ultimately inhibits the proliferation and migration of vascular endothelial cells (87, 88). It is reported that SIRT1 deacetylation at K14 and K20 of PH domain is necessary for binding of Akt to PIP3 and further activation during tumor angiogenesis (89, 90). Several reports suggest that, SIRT1 deacetylate eNOS, stimulate its activity and enhance NO production and tumor angiogenesis (91, 92). Also, FOXO1 and FOXO3 have been reported to repress eNOS, suggesting a link between SIRT1, FOXO and eNOS (93). Increase in SIRT1 deacetylase activity and a consecutive HIF2α activity in ECs, results in acidification and reprogramming toward glutamine metabolism during induction of angiogenesis (94, 95). Studies by Kunhiraman et al. and Edatt et al. reveal that glycolytic inhibition using 2-DG at a sublethal concentration increased the expression and activity of SIRT1, causing reduced expression of angiogenesis associated genes like VEGF and MMP9 (96, 97). Contrary to these reports, Portmann et al., Li et al., and Suzuki et al., report that SIRT1 and VEGF expression is positively correlated during hypoxia induced angiogenesis in breast cancer and lung cancer (98–100). It therefore appears that, the SIRT-1 mediated regulation of angiogenesis and factors regulating it, is largely context dependent.

Like SIRT1, SIRT2 also deacetylate proteins like α-tubulin and histones, being co-localized with tubulin (101–103). Hu et al., demonstrated that SIRT2 knockdown prevented STAT3 phosphorylation and translocation to nucleus, thus decreasing the secretion of VEGF (104, 105). In addition, SIRT2 is reported to directly interact with β-catenin thereby altering the expression of genes like MMPs during tumor angiogenesis (106, 107). Also, it is observed that, SerRS (seryl-tRNA synthetase) plays tumor suppressor and anti-angiogenic role by collaborating with SIRT2 to antagonize c-Myc, a known angiogenic and oncogenic gene (108). Another class of sirtuins, SIRT3 is reported to mediate deacetylation of histones, regulate the stability of tubulin polymers (44, 109, 110), mediate induction of uncoupling protein−1 and regulate Acetyl-CoA synthetase activity (111, 112). Contrary to SIRT1 and SIRT2, SIRT3 is reported to have an opposing effect on angiogenesis, as loss of SIRT3 in human breast cancers, resulted in the upregulation of HIF-1α target genes like VEGF and genes involved in glycolysis (113, 114). Interestingly, it was observed that, SIRT3 overexpression reduced angiogenesis by negatively regulating ROS production, glycolysis as well as HIF-1α stabilization, ultimately resulting in a negative regulation of Warburg effect (115). SIRT4, 5, and 6 has been majorly reported to carry out ADP-ribosylation, desuccinylation and demalonylation rather that deacetylation (34, 116–118). ADP-ribosylation, regulate the activity of glutamate dehydrogenase and PARP (119, 120). Studies from our lab and others has demonstrated that PARPs can regulate the VEGF/VEGFR2 signaling circuit by either transactivation of VEGFR2 or poly ADP ribosylating VEGF to reduce its activity (7, 96, 121). Desuccinylation by SIRT5 suppresses the activities of pyruvate dehydrogenase complex and succinate dehydrogenase (117) leading to the accumulation of succinate and mitochondrial reactive oxygen species, thereby activating HIF1α (122). SIRT5 can also cause desuccinylation and negative regulation of S100A10, a protein that regulate invasion and motility (123). Generally, NAD^+^ levels influence the secretion of various cytokines by inflammatory cells (124). It was found that SIRT6 over expression in pancreatic cancer cells increased TNFα and IL8 production through ADP-ribosylation mediated Ca^2+^ responses (125) and elevated levels of IL8 led to local inflammation, angiogenesis, and EMT (126). Interestingly, Kawahara *et al*. demonstrated SIRT6 interaction with RELA subunit of NFκB to regulate the expression of its target genes involved in tumor progression through deacetylation of promoter region (127, 128). SIRT6 is also reported as a corepressor of HIF1α by deacetylating H3K9 causing downregulation of the expression of genes involved in energy metabolism (129). Furthermore, it is observed that SIRT7 can inhibit HIF1α through a mechanism that is independent of its catalytic activity and regulate the expression of downstream genes like VEGF A and erythropoeitin (130). Also, downregulation of SIRT7 during breast cancer lung metastasis, caused activation of TGFβ signaling pathway and angiogenesis (131). Contrary to this, SIRT7 has been reported to promote angiogenic response by modulating endothelial cell function and VEGF like growth factor expression in mice (132). Altogether these contradicting roles played by sirtuins in tumorigenesis and angiogenesis, highlights the epigenetic regulations involved and unravels the therapeutic potential of sirtuin modulators in treatment of tumor progression by targeting tumor angiogenesis (Table 1).

**Table 1:** The substrates and pathways regulated by different classes of sirtuins.

**Sirtuin**	**Enzyme activity**	**Substrates**	**Pathway Regulated**	**References**
SIRT1	Deacetylase	Histone, p53, FOXO, Rb, p300, PPARγ, NF-κB, PGC-1α, UCP2, MnSOD, Acetyl-CoAsynthetase 1, Smad7, eNOS	Cell survival, metabolism regulation, lifespan regulation, inflammation, oxidative stress response	(14, 46, 133–142)
SIRT2	Deacetylase	α-tubulin, Histone, FOXO, β-catenin	Cell cycle regulation, nervous system development	(101–103, 107)
SIRT3	Deacetylase	Histone, FOXO3a, Acetyl-CoA synthetase2, MnSOD	Regulation of mitochondrial metabolism, ATP-production fatty acid oxidation	(83, 111, 112, 143)
SIRT4	ADP- ribosyl transferase/Deacylase	Glutamate dehydrogenase	Regulation of mitochondrial metabolism, insulin secretion	(29, 144)
SIRT5	Deacetylase Demalonylase Desuccinylase	Cytochrome c, Carbamoyl phosphate synthetase 1	Apoptosis, urea cycle, regulation of protein-protein interaction, protein stability	(32, 34, 37, 38, 145)
SIRT6	Deacetylase /Deacylase ADP-ribosyl-transferase	Histone, HIF1α, TNF-α, NFκB	Genome stability, DNA repair	(29, 35, 118, 127)
SIRT7	Deacetylase	Histone, p53	Regulation of rRNA transcription, cell cycle regulation	(130, 146)

## Post Transcriptional Regulation of Sirtuins: Importance of miRNAs in Tumor Angiogenesis

Along with cytokine and transcriptional factor mediated regulation, precise and effective post transcriptional level regulation are also employed by sirtuins through RNA binding proteins (RBPs) and small non-coding RNA molecules. Micro RNAs are a group of small non-coding RNAs, known as the micro regulators of gene expression. For e.g., miR-34a has been reported to retard endothelial progenitor cell (EPC) mediated angiogenesis by targeting SIRT1 and thereby elevating the levels of acetylated FOXO1, leading to endothelial cell (EC) senescence and cell cycle arrest (147, 148). Similarly, miR-217 has been reported to induce senescence of ECs by modulating the levels of acetylated FOXO1 in a SIRT1 dependent mechanism (149). However, miR-217 has also been reported to promote angiogenesis of Human cytomegalovirus infected endothelial cells by inhibiting SIRT1 and FOXO3A (150). Further, report from our group suggests that miR-106a regulates the expression of MMP9 during cell migration by directly targeting SIRT1 mRNA (151). Our group has also reported that the horizontal transfer of miR-23a from tumor cell colonies can induces angiogenesis by targeting SIRT1 in the recipient endothelial cells (152). Further, miR-212 has been reported to exhibit anti-angiogenic properties, by targeting SIRT1 and Gab1 in endothelial cells (153). SIRT1 has also been reported to inhibit the anti-angiogenic- Notch signaling pathway (54). In addition, TGFβ mediated suppression of SIRT1 expression leading to the activation of Notch signaling pathway in ECs was reported to be depended partly on miR-212 (153). Further, a key micro regulator of angiogenesis and hypoxia responses, miR-138-P_5P has been reported to target SIRT1 (154–156).

## Conclusion: Future Perspectives and Novel Therapeutic Approaches

**Figure 1:**
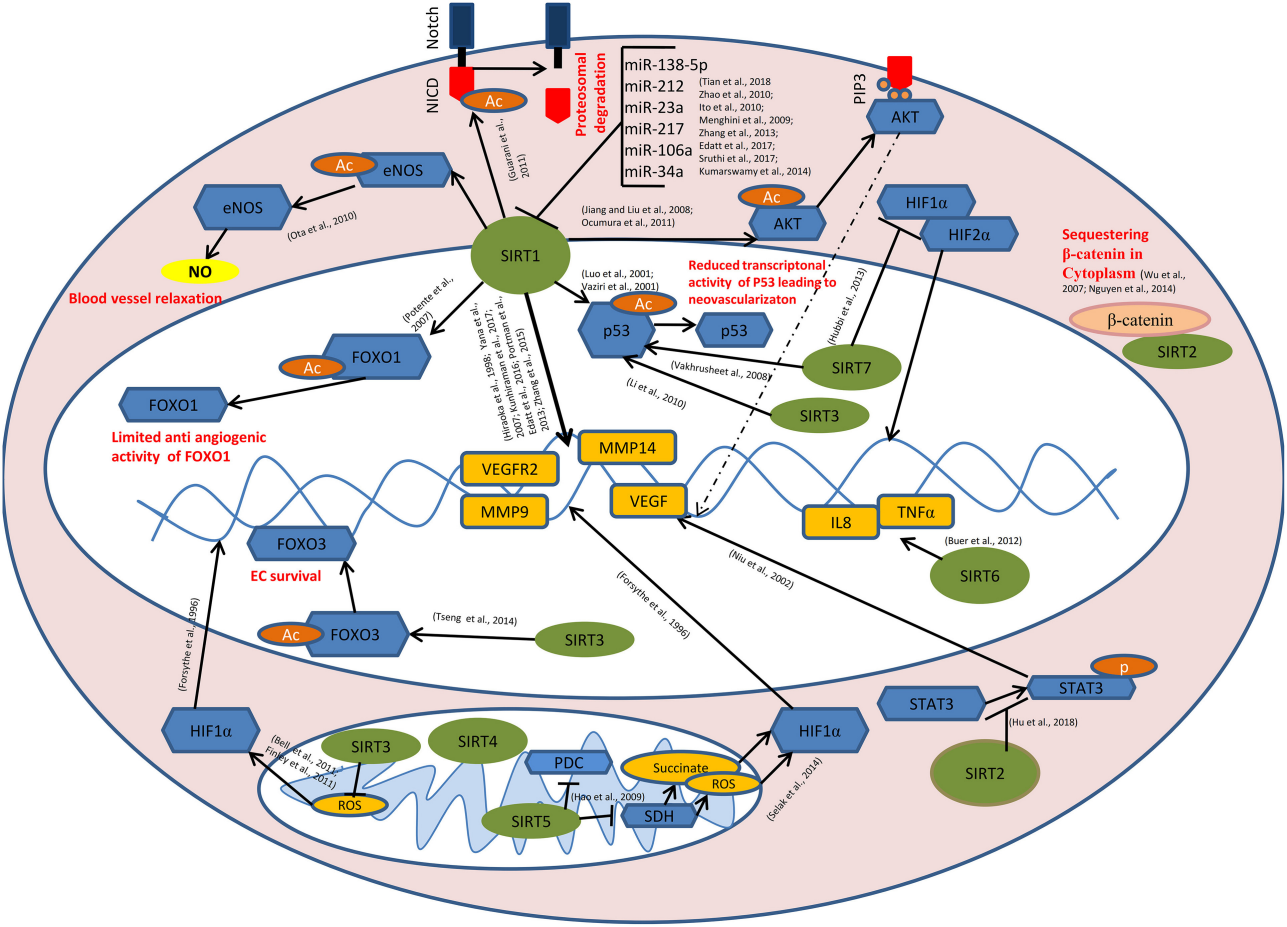
Role of sirtuins in Tumor Angiogenesis: SIRT1 mediates deacetylation of FOXO1, p53, AKT, eNOS, and the intra cellular domain of the Notch protein (NICD) leading to the reduced anti-angiogenic activity of FOXO1, reduced transcriptional activity of p53, induction of AKT signaling causing the transcriptional activation of pro angiogenic genes, enhanced endothelial NO production causing blood vessel relaxation and disassembly followed by the proteasomal degradation of Notch protein respectively. SIRT1 also modulates the expression of VEGF, VEGFR2, MMP9, MMP14, etc. directly by its histone deacetylase activity. miR-34a, miR-106a, miR-217, miR-23a, miR-212, and miR-138-5p targets SIRT1 at post transcriptional level. SIRT3 and SIRT7 catalyze the deacetylation of p53. SIRT7 inhibits HIF-1α stabilization and hence its nuclear translocation. Binding of SIRT2 with β-catenin leads to the sequestration of β-catenin in the cytoplasm, causing modulation in the expression of β-catenin responsive genes including MMPs. SIRT6 mediates the transcriptional activation of IL8 and TNFα which, in turn positively modulates tumor angiogenesis. SIRT2 inhibits STAT3 phosphorylation and its nuclear translocation. SIRT5 inhibits pyruvate dehydrogenase complex (PDC) and succinate dehydrogenase (SDH) causing the accumulation of succinate and reactive oxygen species (ROS) in the mitochondria, leading to HIF-1α activation. SIRT3 negatively regulates mitochondrial ROS production and hence HIF-1α stabilization. SIRT3 mediates deacetylation of FOXO3, thereby promoting endothelial cell (EC) survival under hypoxia. 
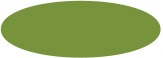
 - Sirtuins, 
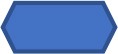
 - transcription factors/enzymes/signaling molecules, 
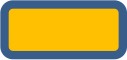
 - downstream genes, 
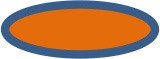
 - acetyl(Ac)/phosphate(p) group, 
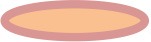
 - β-catenin, 
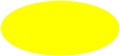
 - Nitric oxide, 
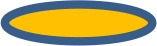
 - Succinate/reactive oxygen species (ROS).

During the past decade, sirtuins have emerged as critical regulators of endothelial cell behavior and have been directly linked to tumor angiogenesis through multiple signaling pathways and cross-talks (Figure 1). Lack of long-term therapeutic efficacy of current anti-angiogenic strategies requisite for novel angiogenesis inhibitors targeting sirtuins (157, 158). Recent discoveries suggest that employing sirtuin isoform specific modulators is a potent anti-angiogenic strategy. Endothelial microparticles enriched with *Sirt6* mRNA induces EC angiogenesis, increases eNOS phosphorylation and prevents release of inflammatory chemokines in diabetic patients (159). Novel approaches like employing various metal (160–162) and inorganic NPs (163–165) have been reported to modulate angiogenesis. Many studies revealed that the shape, size and surface charge of the nanoparticles plays a crucial role in their angiogenic behavior (166, 167). Recently our group has reported that carbon-based nanoparticles (carbon quantum dots) with size <6 nm, inhibit angiogenic process and significantly reduce the expression level of VEGF, VEGFR2, and FGF (168). SirtuinsNano-particle based phytochemicals are reported to regulate sirtuins in cardioprotective treatment strategies (169). So far, no reports are available on the direct correlation with nano particles targeting sirtuins in tumor angiogenesis. Mechanistic studies are under progress on the development of NPs targeting sirtuins and further, tumor angiogenesis. Future studies that unveil the role of potent sirtuin modulators like CQDs at the crossroads of tumor angiogenesis will provide insights for designing novel anti-angiogenic therapies targeting sirtuin.

## Author Contributions

LE and VK contributed to conception and manuscript writing. VR, GR, and SS searched the literature. AP collected data and designed the scheme for the regulation of tumor angiogenesis by sirtuins. LE and VK participated in its coordination and modification. All the authors have read and approved the final manuscript.

### Conflict of Interest

The authors declare that the research was conducted in the absence of any commercial or financial relationships that could be construed as a potential conflict of interest.
